# Regulation of stanniocalcin‐1 secretion by BeWo cells and first trimester human placental tissue from normal pregnancies and those at increased risk of developing preeclampsia

**DOI:** 10.1096/fj.201902426R

**Published:** 2020-03-12

**Authors:** Naila Abid, Joan Embola, Zoe Tryfonos, Julia Bercher, Sandra V. Ashton, Asma Khalil, Baskaran Thilaganathan, Judith E. Cartwright, Guy S. Whitley

**Affiliations:** ^1^ Centre for Vascular Biology, Molecular and Clinical Sciences Research Institute St George's University of London London UK; ^2^ Fetal Medicine Unit St George's University Hospital NHS Foundation Trust London UK

**Keywords:** first trimester, hypoxia, placenta, stanniocalcin‐1, trophoblasts

## Abstract

Stanniocalcin‐1 (STC‐1) is a multi‐functional glycosylated peptide present in the plasma of healthy women postpartum and increased further in pregnancies complicated by preeclampsia. Although the *STC‐1* gene is expressed by the placenta what regulates its secretion and from which cells at the feto‐maternal interface is unknown. Here, we demonstrate for the first time that the syncytiotrophoblast and cytotrophoblast are a major site of STC‐1 protein expression in first trimester placental tissue. Further, in response to low oxygen, first trimester chorionic villous tissue from pregnancies at increased risk of developing preeclampsia secreted significantly more STC‐1 than normal tissue under the same conditions. Using the human trophoblast cell line BeWo we have shown that low oxygen increased the secretion of STC‐1 but it required co‐stimulation with the Adenosine‐3', 5'‐cyclic monophosphate (cAMP) analogue, 8‐Bromo adenosine‐3', 5'‐cyclic monophosphate cAMP (8 Br‐cAMP) to reach significance. Inhibition of Hypoxia inducible factor 2α (HIF‐2α) and the Phosphatidylinositol‐3 kinase (PI_3_‐Kinase)/AKT/Serum and glucocorticoid‐induced kinase‐1(SGK‐1) pathway resulted in significant inhibition of STC‐1 secretion. As both low oxygen and cAMP are known to play a central role in placental function, their regulation of STC‐1 points to a potentially important role in the maintenance of a normal healthy pregnancy and we would hypothesize that it may act to protect against prolonged placental hypoxia seen in preeclampsia.

**Abbreviations**
8 Br‐cAMP: 8‐Bromo adenosine‐3', 5'‐cyclic monophosphate
BSA: bovine serum albumin
cAMP: adenosine‐3', 5'‐cyclic monophosphate
DAB: 3,3′‐diaminobenzidine
DP44mT: di‐2‐pyridylketone‐4,4,‐dimethyl‐3‐thiosemicarbazone
Epac: exchange proteins directly activated by cAMP
FCS: fetal calf serum
GSK‐3β: glycogen synthase kinase‐3β
hCG: human chorionic gonadotrophin
HIF: hypoxia‐Inducible factor
HRE: hypoxia response element
IGF: insulin‐like growth factor
IGFBP4: insulin‐like growth factor binding protein
mTOR: mammalian target of rapamycin
mTORC: mammalian target of rapamycin complex
NDRG‐1: N‐myc downstream‐regulated gene 1
NHS: National Health Service
PAPP‐A: pregnancy‐associated plasma protein‐A
PBS: phosphate‐buffered saline
PBST: phosphate‐buffered saline with tween
PI_3_‐Kinase: phosphatidylinositol‐3 kinase
PKA: protein kinase A
SGK‐1: serum and glucocorticoid‐induced kinase‐1
STC‐1: stanniocalcin‐1
TBS: tris‐buffered saline
TBST: tris‐buffered saline with tween

## 1 INTRODUCTION

Stanniocalcin is a secreted glycoprotein first identified in bony fish where it acts to regulate calcium and phosphate homeostasis.^1^ Although the mammalian homologue stanniocalcin‐1 (STC‐1) may serve a similar function, a wider expression in both normal and pathological tissue would suggest other roles. Support for this in mammals comes from studies indicating different sub‐cellular locations and the expression of different molecular forms.^2^ STC‐1 has been implicated in a spectrum of biological processes including cell migration, apoptosis, and oxidative phosphorylation. It has anti‐inflammatory properties^3^ and acts as a calcium channel blocker in the heart.^4^, ^5^, ^6^, ^7^ STC‐1 is present in the serum of healthy pregnant but not non‐pregnant women and is further elevated in pregnancies complicated by preeclampsia.^8^ Although the expression of the gene is detected in placental tissue and peaks mid‐gestation, the cellular origin was not reported.^8^ Collectively these results suggest that STC‐1 not only has a role in maintaining a healthy pregnancy but may also play a part in the pathology of common pregnancy complications. However, whether it is playing a protective or pathological role has not been established.

One possible role for STC‐1 is inhibiting the activity of pregnancy‐associated plasma protein‐A (PAPP‐A), a proteolytic enzyme that cleaves the inhibitory binding protein IGFBP4 and prevents the release of active IGF‐1.^9^ Circulating maternal IGF‐1 acts to both reduce blood pressure and stimulate fetal growth.^10^ It has also been proposed that the effects of STC‐1 on the kidney could account for the proteinuria and renal failure that can accompany preeclampsia.^8^, ^11^

The aims of this study are threefold: (a) to determine the cellular origin of STC‐1 in the placenta, (b) to investigate how the secretion of STC‐1 is regulated, and (c) to determine whether first trimester placental tissue from the pregnancies at increased risk of developing preeclampsia secrete more STC‐1 than tissue from low‐risk pregnancies.

## 2 MATERIALS AND METHODS

The pharmacological modulators used in this study were obtained from the following manufacturers: 8‐Bromo adenosine‐3', 5'‐cyclic monophosphate ( 8‐Br‐cAMP), sodium salt (Biolog, Germany), protein kinase A (PKA) inhibitor, N‐[2‐(p‐bromocinnamylamino) ethyl]‐5‐isoquinoline sulfonamide (H89), the Exchange proteins directly activated by cAMP (Epac) inhibitor, α‐[(2‐(3‐Chlorophenyl)hydrazinylidene]‐5‐(1,1‐dimethylethyl)‐β‐oxo‐3‐isoxazolepropanenitrile (ESI‐09), the mTORC 1 and 2 inhibitor, KU0063794 and the SGK‐1 inhibitor, GSK650394 (Tocris, Abingdon UK), PT2385 (AbCam Cambridge UK), the PI_3‐_Kinase inhibitor LY294002 (Sellekchem, Absource Diagnostics, Germany), AKT inhibitor IV, and Rapamycin (Calbiochem, Watford UK), Desferrioximine and NDRG‐1 activator, di‐2‐pyridylketone‐4,4,‐dimethyl‐3‐thiosemicarbazone (DP44mT; Sigma Gillingham, UK).

### 2.1 Doppler ultrasound scanning of first trimester uterine arteries

Doppler ultrasound screening of uterine arteries was performed on women undergoing elective surgical termination of pregnancy at St George's University Hospital, NHS Foundation Trust. Inclusion criteria included singleton pregnancy, gestational age 8‐14 weeks, normal fetal anatomy and nuchal translucency thickness, and no known maternal medical condition or history of recurrent miscarriage. The gestational age was calculated by crown‐rump length measurement. Doppler ultrasound was performed by a trained sonographer as described previously.^12^ Following a study of 10 000 ongoing pregnancies, we have established reference ranges of the resistance indices.^13^ In this study, a high‐ resistance index (RI) is defined as a pregnancy with bilateral uterine artery notches and a mean RI ≥ 95th centile; while a normal‐RI presents with no uterine artery notches and a mean RI < 95th centile. The risk of developing preeclampsia would be five times greater in the high‐RI group had the pregnancy gone to term.

### 2.2 Serum samples obtained during the third trimester

The patients were recruited from the antenatal clinics and the inpatient wards at St George's Hospital. There was no significant difference in gestational age between the normotensive and pre‐eclamptic pregnancies. The diagnosis of preeclampsia was made according to the criteria of the International Society for the Study of Hypertension in Pregnancy.^14^ The exclusion criteria included major fetal abnormalities and those ending in termination, miscarriage, or fetal death before 24 weeks. Data on maternal baseline demographics and pregnancy outcomes were collected from the hospital maternity records.

### 2.3 Immunohistochemistry

Paraffin‐embedded placental tissue sections (7 µm) were cut and mounted on slides and immersed in xylene, twice for 5 mins. The slides were then re‐hydrated using a series of ethanol dilutions from 100% to 70% (v/v) and then washed in water for 5 mins. Heat inactivating epitope retrieval of STC‐1 and cytokeratin‐7 (CK‐7) was performed using 11 mM tri‐sodium citrate, pH 6.0 containing 0.05% (v/v) Tween20 or 10 mM Tris buffer pH 10, for the CK‐7, respectively.

Sections were washed twice with Tris‐buffered saline pH 10 [(TBS) containing 140 mM NaCl, 2.68 mM KCL, 16.7 mM Trisma base] and permeabilized using TBS with 0.2% (v/v) Triton X‐100 for 5 min. Sections were washed twice and non‐specific binding blocked using TBS containing 1% (w/v) bovine serum albumin (BSA), or 10% (v/v) goat serum for one h at room temperature (RT). Sections were then incubated with a polyclonal mouse anti‐human STC‐1 antibody (1 µg/mL) (SantaCruz, California, USA) or polyclonal mouse anti‐human CK‐7 antibody [0.621 µg/ml) (Dako, Glostrup Municipality, Denmark) in TBS containing 1% (v/v) BSA, 0.025% (v/v) Triton X‐100 in a humidified chamber overnight. Non‐immune IgG of equal concentrations from the same species were used as negative controls.

After an overnight incubation, sections were washed with TBS and endogenous peroxidase activity blocked with 0.3% (v/v) H_2_0_2_ in TBS. Sections were then incubated with Histostain broad spectrum biotin/streptavidin system (Invitrogen, Waltham, Massachusetts, USA) following the manufacturer's protocol, washed twice in TBS before being incubated with 2‐Solution (3,3′‐diaminobenzidine) DAB kit (Roche, Basel, Switzerland). Sections were counter stained using Harris Haematoxylin solution and the nuclei blued using saturated lithium carbonate solution. Images were taken using an Olympus IX70 microscope with an attached XC10 camera (Olympus, Tokyo, Japan) using CellSens Dimensions software (Olympus).

### 2.4 Immunocytochemistry

Cells were grown on gelatine‐coated glass coverslips under the appropriate conditions and then washed twice in PBS before fixing with ice–cold methanol for 10 min. The cells were then washed twice for 5 min with PBS and incubated in PBS containing 0.2% (v/v) Tween 20. After 10 min the cells were washed twice for 5 min with PBS and blocked with 10% (v/v) goat serum in PBS for 20 mins at room temperature. The fixed and blocked cells underwent three further 5 min washes in PBS and were incubated with either the primary antibody (Anti‐STC1 MAB2958 5 µg/mL; R&D Systems) or isotype‐matched immunoglobulin control (IgG2βκ, ebioscince) for 1 h at room temperature. Cells were then washed thrice in PBS for 5 min and incubated in the secondary antibody (anti‐mouse biotinylated IgG; Vector Labs) for 45 min at room temperature. Following, three washes with PBS for 5 min, cells were incubated with streptavidin‐fluorescein (3.3µg/ml, Vector Labs) for 30 min in the dark, at room temperature. All antibodies were diluted in PBS containing 1.5% (v/v) GS and 0.2% (v/v) Tween‐20. Cells were washed thrice with PBS and 1‐2 drops of DAPI‐containing Vectashield (Vector Labs) was added. Images were captured using a Nikon A1R Confocal Microscope and imported into ImageJ. Each image was adjusted using the Brightness and Contrast tool using the identical parameters and saved as.PNG.

### 2.5 Cell culture

The choriocarcinoma‐derived cytotrophoblast cell line (BeWo) used in this study were maintained in Dulbecco's Modified Eagle's Medium (with 4500 mg/L glucose, sodium pyruvate and sodium bicarbonate, Sigma) and Ham's F12 medium (1:1) supplemented with 10% (v/v) Fetal Calf Serum (FCS), penicillin (100 IU/mL), streptomycin (100 µg/mL), and l‐glutamine (2 mmol/L).

### 2.6 Stimulating cells for enzyme linked immunosorbent assay analysis

BeWo cells were seeded at a density of 4‐5 × 10^5^ cells per well of a 6 well plate in 1.5 mL of the DMEM/HAM’s F‐12 containing 10% (v/v) FCS. The cells were incubated overnight to allow them to settle and then incubated according to the experiments with 100 μM 8Br‐cAMP, pharmacological inhibitors (GSK650394 at both 100 and 10 μM, LY294002 at 50 μM and 10 μM, AKT inhibitor IV at 2.5 μM, 5 μM KU0063794 at 1 μM, Rapamycin at 10 ng/mL) and also stimulators (DP44mT at 25 and 10 μM). To assess the effect of reduced oxygen, cells were placed inside sealed blood bags (Baldwin Medical Supplies, Knoxville, VIC, Australia) flushed with 1% oxygen (O_2_) with 5% CO_2_ in N_2_ at 37°C as previously described.^15^ Otherwise cells were incubated at 5% CO_2_ in air at 37°C in a humidified incubator. All the cells were incubated for 48 hours at 37°C.

### 2.7 Determination of STC‐1 in the culture medium

After incubation, the cell culture medium was collected, centrifuged at 14 000 g for 5 min and the resulting supernatant separated from the pellet. The volume of the supernatant was determined. STC‐1 in the culture supernatant was determined by enzyme‐linked immunosorbent assay (ELISA) as per the manufacturer's instructions (DuoSet R&D Systems). The cells were washed once with PBS and lysed, the protein concentration was determined using the Bradford protein assay. The amount STC‐1 produced was then expressed per mg of total cellular protein.

### 2.8 Stimulating cells for western blot analysis

BeWo cells were seeded at a density of 6 × 10^6^ cells per 10 cm dish in culture media and left to adhere overnight. The cells were then incubated with the appropriate inhibitor for 20 min before the addition of 8Br‐cAMP (100 μM). Cells were either incubated at 21% O_2_, or 1% O_2_, in 5% CO_2_ and air. All cells were incubated for up to 48 h at 37°C.

### 2.9 Western blot analysis for cells and tissue

After incubation the cells were lysed in up to 200 μL of RIPA buffer containing 100 mM sodium orthovanadate, 17 mg/mL aprotinin, phosphoSTOP (Roche) and 10 mg/mL phenylmethylsulonylfluoride), sonicated, and centrifuged at 14 000 *g* for 5 min and the supernatant collected. Placental tissue (5‐10 mg) was homogenized in 1 mL of RIPA buffer using a FastPrep‐24 homogenization tube containing Lysis Matrix D. Protein concentrations were determined using the Bradford protein assay. Approximately 30‐50 μg of protein per well was resolved on a polyacrylamide gel before transfer to Immobilon‐FL transfer membrane (Millipore, UK). Protein loading and transfer efficiency were followed using tubulin. Non‐specific reactivity was then blocked using Tris‐buffered saline (TBS) with 5% (w/v) low fat milk powder for 1 h at room temperature. Blots were probed with the following antibodies: HIF1α (Cell signalling, 1:1000), HIF2α (Novis, NB100‐122 1:750), and Tubulin (Sigma 1:10 000) in 5% (w/v) BSA in TBS containing 01% (v/v) Tween 20.

The blots were then washed in TBST and incubated with goat anti‐rabbit IgG conjugated to horseradish peroxidase (1:10 000; A5420, Sigma, UK) for 1 h at room temperature, and antigen‐antibody complexes were detected using an enhanced chemiluminescence system (Amersham Biosciences UK). Blots were subsequently stripped in buffer containing 62.5 mM Tris pH 6.7, 2% (w/v) SDS and 100 mM β‐mercaptoethanol and probed with a rabbit polyclonal antibody to human β‐actin or mouse monoclonal antibody to human anti‐Tubulin (1:10 000; Ab7291, Abcam UK). Where indicated, western blots were scanned and the integrated intensity of each band determined using ImageJ (http://rsbweb.nih.gov/ij/docs/intro.html). Results were expressed as a ratio to loading control within the same sample.

### 2.10 Transfection of BeWo cells with siRNA

Cells were cultured in HAM’s‐F12 medium containing 10% (v/v) FCS without antibiotics for 48 h prior to transfection by Nucleofection (Amaxa Biosystems, Germany). Following trypsinzation 2 × 10^6^ cells were resuspended in 100 µl of cell line solution L with either 300nM of control siRNA‐A (Santa Cruz sc‐37007) or targeted si‐RNA EPAS‐1 (Santa Cruz sc‐35316) and transfected following the manufacturers' instructions using programs X‐005 as previously detailed.^16^ Following transfection, cells were seeded at approximately 5 × 10^5^/well of a 6‐well plate in HAM’s‐F12 medium containing 10% (v/v) FCS without antibiotics. After 24 hours, the medium was replaced with DMEM/HAM’s F‐12 containing 10% (v/v) FCS and incubated for 48 h in 1% O_2_ with and without 100 µM 8Br‐cAMP.

### 2.11 Ethics

Subjects involved in this study gave informed written consent and the study protocols had either local Wandsworth Ethics Committee (ref: 01.96.8, 01.78.5) or the London‐Stanmore Research Ethics Committee (ref: 12/LO/0810) approval.

### 2.12 Statistical analysis

Graph Pad Prism 5.0 (Graph Pad Software Inc, San Diego, CA, USA) was used for statistical analysis. At least three independent experiments were performed and the results expressed as mean + standard error of the mean. Where appropriate a D'Agostino & Pearson normality test was performed followed by a one‐way ANOVA with Holm‐Sidak's multiple comparisons test. A *P* value of <.05 was considered to be statistically significant.

## 3 RESULTS

### 3.1 Expression of STC‐1 in placental tissue and BeWo cells

**Figure 1:**
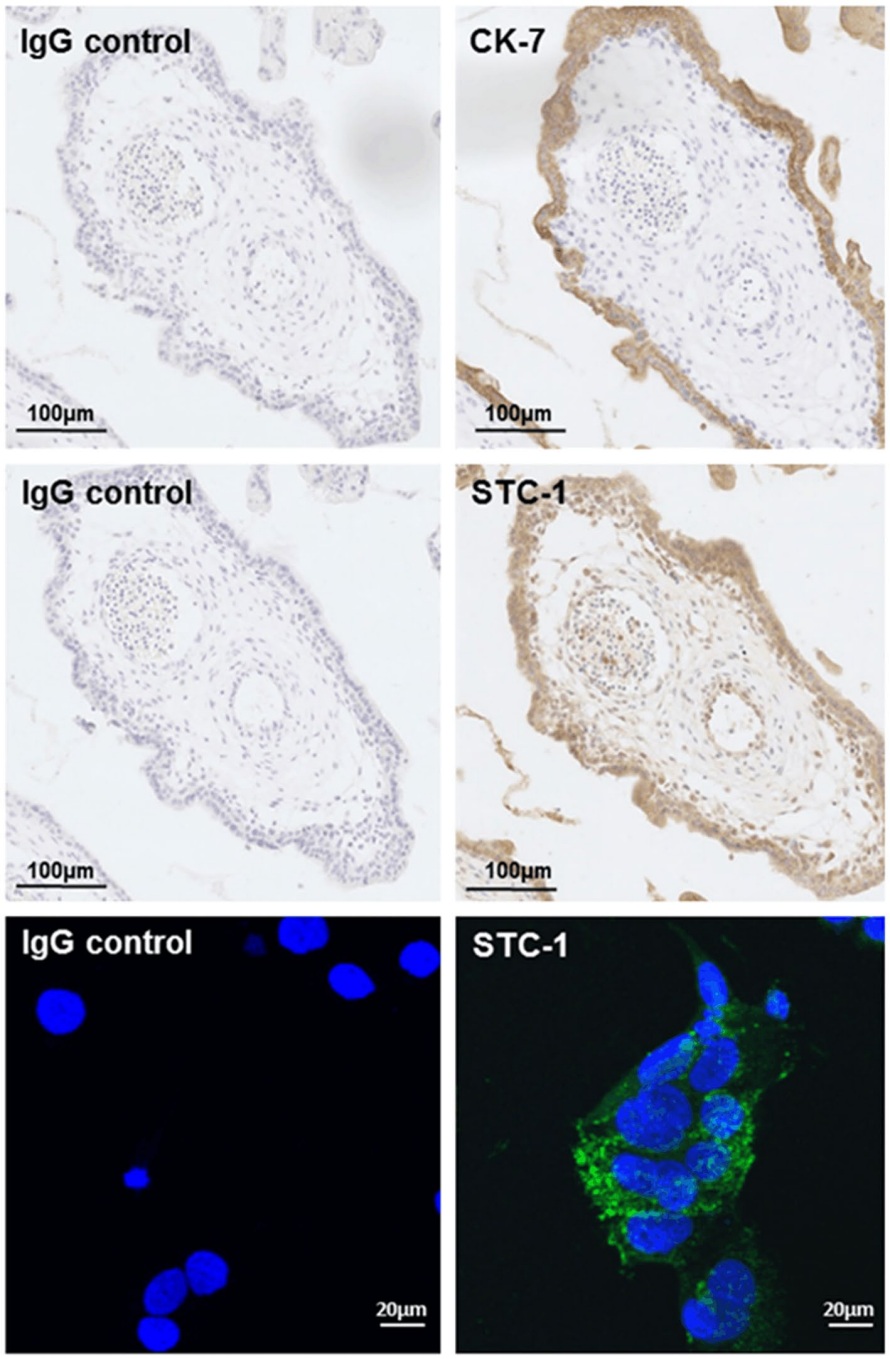
Histological and cytochemical analysis of first trimester placental tissue sections and BeWo cells grown in culture. Top Left: Treated with non‐immune IgG negative control stained purple. Top Right: Treated with mouse monoclonal anti‐CK‐7 antibody, brown highlights positive expression of CK‐7. Middle Left: Treated with non‐immune IgG negative control, Middle Right: Treated with mouse anti‐STC‐1 antibody, brown highlights positive expression STC‐1. The data are representative of results from three different patient samples performed on separate occasions. BeWo cells were grown 1% O_2_ and stimulated with 100 µM 8Br‐cAMP for 24 h. Bottom Left: Treated with non‐immune IgG negative control, Bottom Right: Treated with mouse anti‐STC‐1 antibody (Green) and the nuclei stained with Dapi (Blue)

Immunohistochemical staining of adjacent sections of first trimester chorionic tissue demonstrated co‐incident expression of CK‐7 and STC‐1 in both the syncytium and underlying cytotrophoblasts (Figure 1). Some staining in the placental endothelial cells and stromal cells was observed. Immunocytochemical analysis of BeWo cells stimulated with 100µM 8Br‐cAMP and incubated in 1% O_2_ for 48 h indicated largely cytoplasmic staining for STC‐1 (Figure 1).

### 3.2 Serum STC‐1 in normal pregnancies and pregnancies complicated by preeclampsia

**Figure 2:**
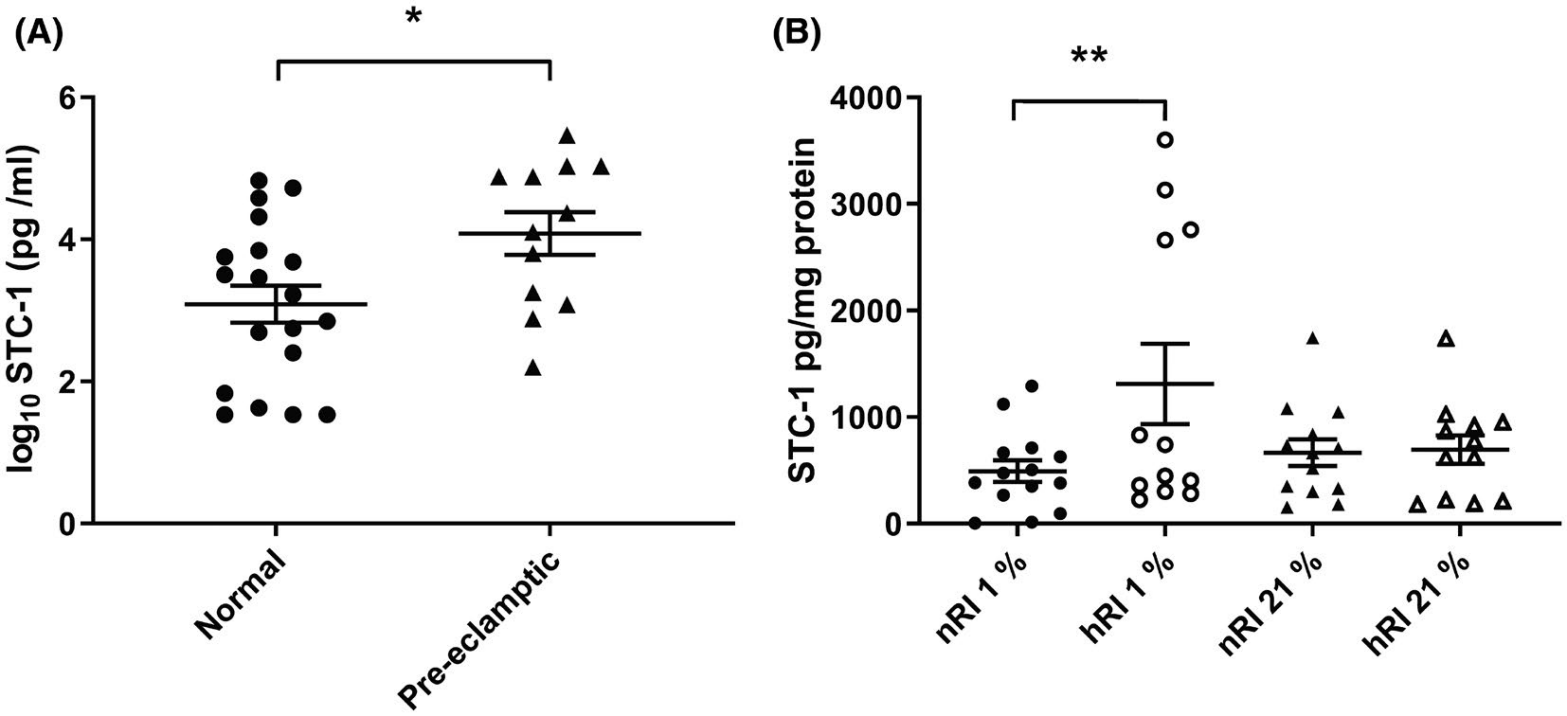
Concentration of STC‐1 in serum obtained from pregnancies in the third trimester with known outcomes. A, Serum was obtained from normal and preeclamptic pregnancies the third trimester. The concentration of STC‐1 was determined by ELISA and the data log‐transformed. The data shown are expressed as mean ± SEM of n = 19 normal pregnancies and n = 11 preeclamptic pregnancies. Significance was determined using an unpaired student t test *P < .05. Secretion of STC‐1 by first trimester chorionic tissue from nRI and hRI pregnancies incubated at 1% and 21% O_2_. B, First trimester chorionic villous tissue was incubated at either 1% or 21% O_2_ for 72 h_._ The medium was then collected and the tissue weighed. STC‐1 was determined by ELISA and the amount produced was corrected for the weight of tissue. The number of pregnancies in each group were nRI 1% (n = 14), hRI 1% (n = 12), nRI 21% (n = 13) hRI 21% (n = 12). The data were tested for normality using the D'Agostino & Pearson normality test before analysis using a one‐way ANOVA followed by a Holm‐Sidak's multiple comparisons test, **P < .01

The circulating serum concentration of STC‐1 at term in healthy pregnant women and women with preeclampsia was determined by ELISA. The concentration of STC‐1 in normal pregnancy was 11.36 ng/mL ± 4.8 SEM (n = 19) and 63.71 ± 34.5 SEM ng/mL (n = 12) for those pregnancies complicated by preeclampsia, *P* < .05 (Figure 2A).

### 3.3 Secretion of STC‐1 by placental tissue

Freshly isolated first trimester placental tissue from normal‐RI and high‐RI pregnancies were incubated for 24 h at 1% O_2._ They were then transferred to fresh medium and cultured for a further 72 h. There was significantly more STC‐1 secreted from the tissue from the high‐RI pregnancies compared to the normal‐RI placental tissue (*P* < .01; n > 12 experiments as determined by ELISA; Figure 2B).There was no significant difference in the secretion of STC‐1 between the two groups when tissue was cultured at 21% O_2._

### 3.4 The effect of 8Br‐cAMP and low O_2_ on the secretion of STC1 by the trophoblast cell line BeWo

**Figure 3:**
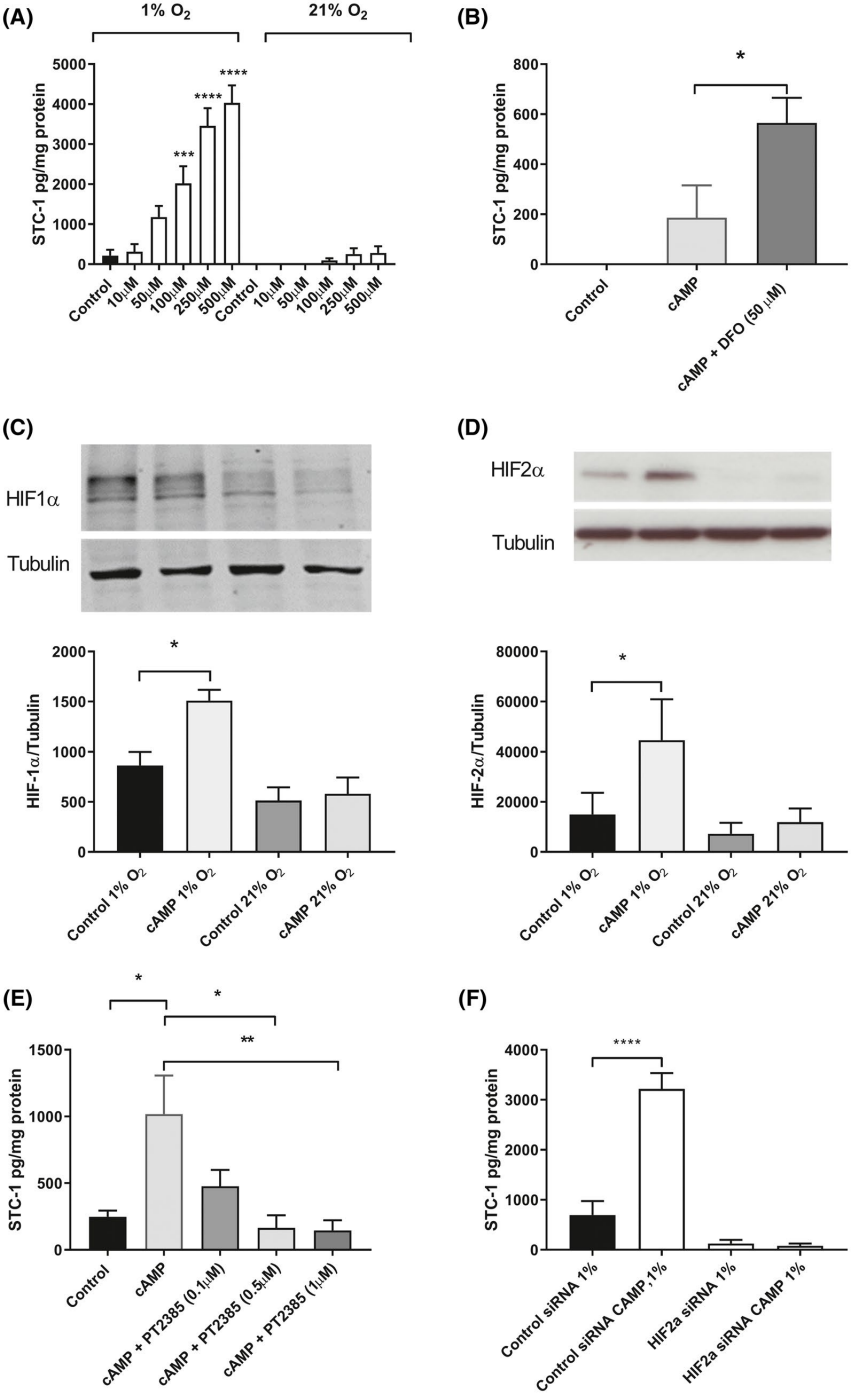
The effect of cAMP and oxygen on production of STC‐1 by BeWo cells. A, BeWo cells (3 × 10^5^ per well) were incubated in either 1% or 21% oxygen with increasing concentration of 8Bromo‐cAMP. The data were expressed as mean + sem, of n = 4 independent expts. B, BeWo cells were cultured in 21% O_2_ and stimulated with 100 µM 8 Bromo‐cAMP in the presence and absence of 50 µM DFO (n = 4 independent expts). The effect of the low oxygen and cAMP on the expression of HIF‐1, and HIF‐2α. BeWo cells were cultured in 1% and 21% O_2_ in presence and absence of 100 µM 8 Bromo‐cAMP for 48 h and cell lysates were examined by western blot analysis. C, HIF‐1α (n = 4 independent expts) and D, HIF‐2α (n = 7 independent expts) were detected by chemiluminescence, using tubulin as the loading control. The effect of inhibiting HIF‐2α on the secretion of STC‐1 was determined using (E) PT2385, (n = 3 independent expts) or (F) RNA interference, (n = 4 independent expts) in the presence and absence of 100 µM 8 Bromo‐cAMP. Stimulation in all experiments was for 48 h. The concentration of STC‐1 in the medium was determined by ELISA. Total cellular protein was determined by Bradford assay. The results are expressed as pg of STC‐1/mg of protein. The level of significance was determined using either, a Student t test or a one‐way ANOVA and a Sidak's multiple comparisons test where appropriate, *P < .05, **P < .01, *P* < .005(**), *P* < .0005 (***), *P* < .0001 (****)

The membrane permeable cAMP analogue, 8Br‐cAMP, promotes formation of syncytial structures and the secretion of Human chorionic gonadotrophin (hCG) by BeWo cells.^17^ We, therefore, examined the effect of cAMP on the secretion of STC‐1 in these cells. At 21% O_2_ the effect of 8Br‐cAMP on the secretion of STC‐1 by BeWo cells did not reach statistical significance. Culturing cells in 1% O_2_ in combination with 8Br‐cAMP lead to an increase in the secretion of STC‐1 compared to 21% O_2_ that reached significance at 100 µM with a ninefold increase (*P* < .001). The highest dose of 500 µM resulted in a 19 fold increase (*P* < .0001). These data indicate that both cAMP and O_2_ regulated pathways act synergistically to stimulate STC‐1 secretion in BeWo cells (Figure 3A). It was also possible to mimic the effect of reducing the oxygen concentration by treating the cells with the iron chelator desferrioximine, which, at 21% O_2_ lead to a threefold increase in the secretion of STC‐1 over 8Br‐cAMP alone and a significant increase compared to control (*P* < .05; Figure 3B).

### 3.5 Role of O_2_ regulated pathways in STC‐1 secretion

Oxygen regulates cellular activity in a number of ways including through the increased stabilization of HIF. Western blot analysis demonstrated the expression of both HIF‐1 and 2α in BeWo cells cultured for 48 h in 1% O_2_ (Figure 3C, D). To determine which factor was involved in the regulation of STC‐1 we used a HIF‐2α specific antagonist, PT2385.^18^ Incubation of BeWo cells with 1 µM PT2385 significantly inhibited the secretion of STC‐1 in response to 1% O_2_/100 µM 8Br‐cAMP (Figure 3E). We confirmed the involvement of HIF‐2α using RNA‐mediated interference. Following transfection with non‐targeting control si‐RNA there was a significant increase in the secretion of STC‐1 following the culture of BeWo cells 48 h in 1% O_2_ and 100 µm cAMP. However transfection with HIF‐2α siRNA inhibited 1% O_2_ and 100 µm cAMP stimulated STC‐1 secretion completely (Figure 3F).

### 3.6 Investigation of cAMP dependent pathways

**Figure 4:**
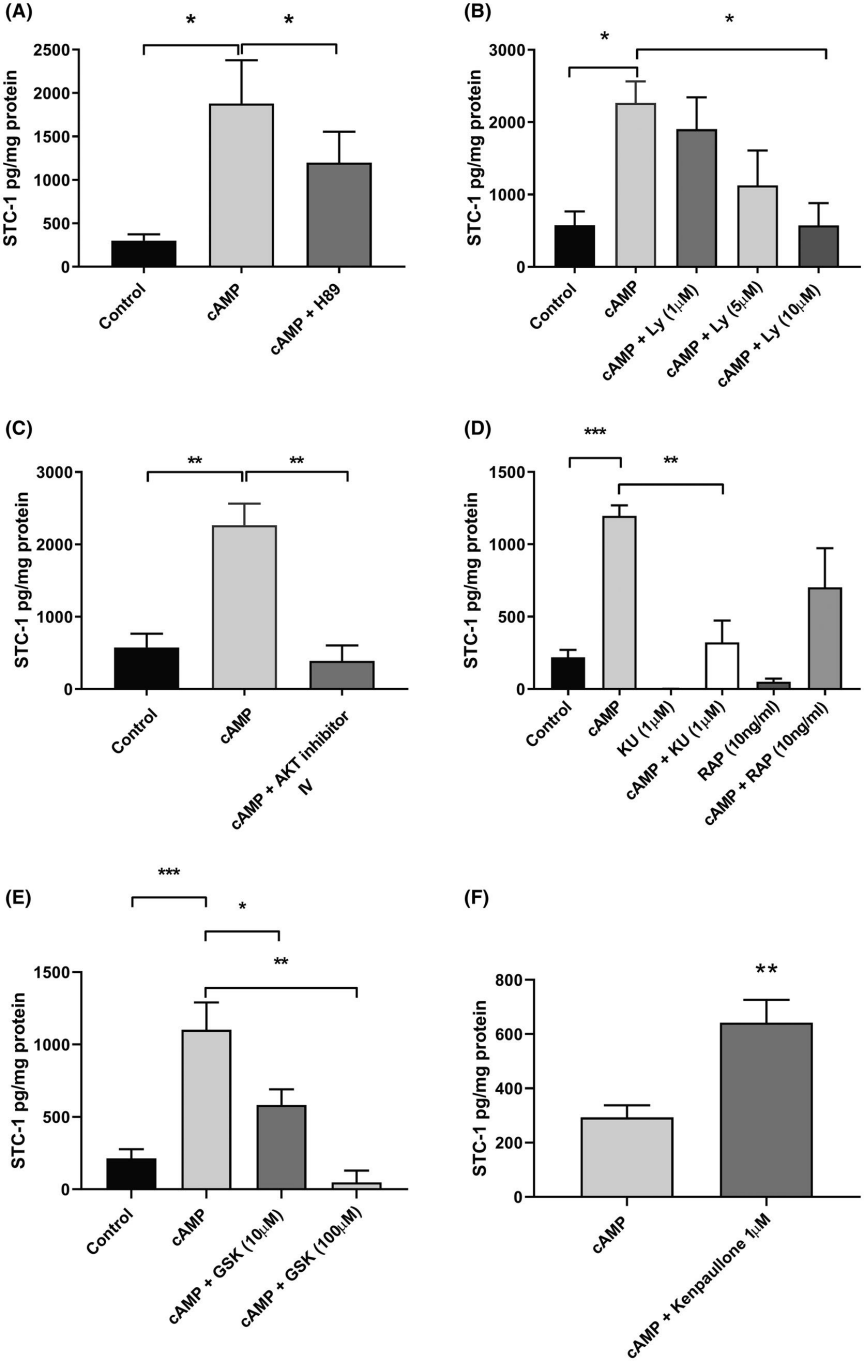
The effect of the inhibiting PI_3_kinase, Akt and mTORC 1 and 2 on the secretion of STC‐1 by BeWo cells. BeWo cells were cultured in 1% O_2_ in 100 µM 8 Bromo‐cAMP in the presence and absence of (A) the PKA inhibitor H89 (10 µM), (B) the PI3kinase inhibitor Ly 294002 (LY) at 1, 5 and 10 µM (n = 3) (C) Akt inhibitor IV (2.5 µM) or (D) either the mTORC1 inhibitor rapamycin (RAP) or the mTORC1 and 2 inhibitor KU 0063794 (KU). (E) 10 µM (n = 5) and 100 µM (n = 3) GSK650394 in 1% O_2_ or, (F) 1 µM Kenpaullone. After 48 h, the medium was removed and the STC‐1 secreted measured by ELISA. Total cellular protein was determined by Bradford assay. The results are expressed as pg of STC‐1/mg of protein. All data were expressed as mean + SEM. The level of significance was determined using a one‐way ANOVA and a Sidak's multiple comparisons test, or an unpaired *t*‐test *P < .5, **P < .01 and ***P < .001

Having established a role for cAMP in the secretion of STC‐1, we investigated downstream targets of cAMP, PKA and Epac. BeWo cells were treated with inhibitors of PKA, H‐89 (10 µM), and the inhibitor of Epac, ESI‐09 (50 and 100 µM) and then stimulated with 1% O_2_/100 µM 8Br‐cAMP and incubated for 48 h. Inhibition of PKA with H89 had a small, but significant effect on STC‐1 secretion (*P* < .05, n = 6; Figure 4A); however, inhibition of Epac had no effect (data not shown).

### 3.7 Role of the PI_3_‐Kinase/AKT pathway in the secretion of STC‐1 by BeWo

There is evidence to suggest that the PI_3_‐Kinase/Akt pathway can be activated in response to elevated cAMP.^19^ To determine whether activation of the PI_3_‐Kinase/Akt pathway is involved in regulating the secretion of STC‐1 by trophoblasts we used the pharmacological modulator Ly294002. Ly294002 inhibited the secretion of STC‐1 in a dose‐dependent manner, reaching statistical significance at 10 µM (*P* < .05; Figure 4B). Inhibiting the activation of AKT with AKT inhibitor IV also significantly inhibited the secretion of STC‐1 in response to 8Br‐cAMP/1% O_2_ to below the levels of 1% O_2_ alone (*P* < .01; Figure 4C).

### 3.8 Role of mammalian target of rapamycin in the secretion of STC‐1 by BeWo

Mammalian target of rapamycin (mTOR) is associated with two complexes, mTOR complex (mTORC)‐1 and ‐2, which act as both downstream and upstream of Akt, respectively. To assess the role of mTOR in the secretion of STC‐1 we used two inhibitors; rapamycin (RAP) which targets mTORC1 and KU‐0063794 which inhibits both mTORC1 and 2. Only KU‐0063794 (KU) had a significant effect on the secretion of STC‐1 (*P* < .01; Figure 4D).

### 3.9 Role of SGK‐1 in the secretion of STC‐1 by BeWo

The above data implicate mTORC2 in the mediation of cAMP/1% O_2_ stimulated STC‐1 secretion. Activation of mTORC2 results in the activation of not only AKT but also a number of other pathways including SGK‐1. Inhibition of SGK‐1 with GSK 650394 significantly reduced the secretion of STC‐1 in a dose‐dependent manner (10 µM *P* < .05 n = 6 experiments, 100 µM *P* < .01; Figure 4E). Downstream targets of SGK‐1 include N‐Myc Downstream‐Regulated Gene 1 (NDRG‐1) and Glycogen Synthase Kinase‐3β (GSK‐3β). Using western blot analysis, we were able to demonstrate an increase in the phosphorylation and therefore activation of NDRG‐1 in response to cAMP/1% O_2_. However, direct activation NDRG‐1 following stimulation with Dp44mt had no effect on the secretion of STC‐1 by BeWo cells (data not shown). Inhibition of HIF‐2α with PT2385 had no effect on the phosphorylation of NDRG‐1 (data not shown). To explore the possible involvement of the GSK‐3β in this process, we incubated BeWo cells with kenpaullone, a GSK‐3β inhibitor which significantly (*P* < .01) enhanced the secretion of STC‐1 in response to cAMP/1% O_2_ (Figure 4F).

## 4 DISCUSSION

The placenta is a pregnancy‐specific organ that develops rapidly throughout gestation. It acts as the crucial interface between the fetus and the mother. It has a number of functions but pertinent to this study is its role as a major endocrine organ, modulating, and adapting the maternal response to pregnancy. We have focused on STC‐1, a little studied hormone, which in health is only detectable in the circulation during pregnancy and is further elevated in pregnancies complicated by preeclampsia.

In the current study, we established that the protein STC‐1 was predominantly expressed in the syncytiotrophoblast and cytotrophoblast cells of first trimester placental tissue where it is co‐localized with the expression of CK‐7. Less intense expression was also seen in placental endothelial and stromal cells. We were further able to demonstrate a significant difference in the circulating concentration of STC‐1 in the women with preeclampsia compared to normal pregnancies. These results were largely consistent with previously published data from plasma collected post‐partum.^8^ In addition, we found that the secretion of STC‐1 by first trimester chorionic villous explants cultured at 1% O_2_ was significantly greater in pregnancies at increased risk of developing preeclampsia. As the syncytium is the likely source of STC‐1 found in the maternal circulation we examined the possible mechanisms that might regulate the secretion of STC‐1 using first trimester placental tissue and the BeWo trophoblast cell line.

Human chorionic gonadotrophin stimulates the differentiation of syncytiotrophoblast through the elevation of intracellular cAMP.^20^ We, therefore, determined whether cAMP could play a role in regulating STC‐1 secretion using the membrane permeable phosphodiesterase resistant analogue, 8Br‐cAMP. Although the effect of cAMP in regulating *STC‐1* mRNA expression has been studied, the effect is tissue specific. In human endometrial stromal cells and rat neuroblastoma cells, cAMP stimulates STC‐1 expression.^21^, ^22^ However, in rat Sertoli and Leydig cells the opposite effect was observed.^23^ It has previously been reported that elevated intracellular cAMP‐induced cell fusion and differentiation in BeWo cells.^24^ In this study, 8Br‐cAMP alone had no effect on the secretion of STC‐1 by BeWo cells cultured in atmospheric oxygen suggesting that differentiation of BeWo cells alone was insufficient for secretion.

In early gestation, the human fetus develops in a relatively low oxygen environment. Around the 10th week of pregnancy, the delivery of blood to developing placenta changes significantly due to the remodeling of the maternal arteries and the loss of the trophoblast plugs.^25^ In pregnancies that later develop preeclampsia and/or fetal growth restriction, this remodeling fails to occur adequately resulting in placental under‐perfusion and intermittent hypoxia. We, therefore, hypothesized that one possible stimulus for the increased placental expression and secretion of STC‐1 seen in pre‐eclamptic pregnancies could be low oxygen. In support of this, low oxygen is associated with increased *STC‐1* gene expression in a number of pathological situations including cerebral ischemia^26^ and carcinogenesis^27^, ^28^, ^29^ and heart failure.^5^ Using a human tumor cell line, both low oxygen and the chemical induction of hypoxia, using either desferoxamine or cobalt chloride, also increased *STC‐1* gene expression.^30^ Culturing BeWo cells in 1% O_2_ did not increase the secretion of STC‐1. We, therefore, examined whether low oxygen in combination with increased intracellular cAMP could act to stimulate STC‐1 secretion and found that there was a dose‐dependent increase in the secretion of STC‐1 in response to 8Br‐cAMP when cultured in low O_2_ which reached statistical significance at 100 µM 8Br‐cAMP. There are at least two intracellular targets for cAMP in trophoblasts, PKA and Epac. Using inhibitors and activators of these targets, we were able to establish that the majority of the stimulus for the secretion of STC‐1 by BeWo cells was mediated by PKA which was in accord with earlier findings in the ovary.^31^

Oxygen‐responsive genes are regulated by members of the HIF family of transcription factors. The best‐studied isoforms are HIF‐1α and HIF‐2α which share a number of structural and functional similarities but differ in expression patterns, target genes, and regulatory mechanisms. In low O_2,_ HIF‐1α and HIF‐2α dimerize with HIF‐1β forming HIF‐1 and HIF‐2, respectively. They then translocate to the nucleus where they regulate target gene expression by binding to and activating a hypoxia response element (HRE). Both HIF‐1α and HIF‐2α proteins are expressed in the human placenta. HIF‐1α is widely distributed, whereas the expression of HIF‐2α is restricted largely to the trophoblasts.^32^, ^33^ A HRE has been identified in the promotor of STC‐1^34^ and using the HIF‐2α specific inhibitor PT2385,^18^ which antagonizes the binding of HIF‐2α to HIF‐1β, we were able to demonstrate that the secretion of STC‐1 by BeWo cells was HIF‐2α mediated. To investigate the effect of cAMP on the expression of HIF‐2α, we used western blot analysis. As predicted, cells grown in low O_2_ expressed HIF‐2α and stimulation of cells with 8Br‐cAMP led to a significant increase in expression. PT2385 is a selective and potent small‐molecule inhibitor of HIF‐2α identified by a structure‐based design approach. It allosterically binds to HIF‐2α and blocks heterodimerization with HIF‐1β.^18^ Crystallographic analysis of this interaction reveals no such binding of PT2385 to HIF‐1α. Although this does not exclude off‐target effects in the current study, it does suggest the effects demonstrated are not mediated through the inhibition of HIF‐1α. The importance of HIF‐2α in regulating the secretion of STC‐1 was confirmed using validated RNA‐interference techniques targeting the expression of HIF‐2α.

Using pharmacological inhibitors and activators, we examined the interaction between HIF‐2α and 8Br‐cAMP further and have shown that inhibiting PI_3_‐kinase/Akt pathway with Ly294002, and Akt‐inhibitor IV in BeWo cells led to significant inhibition of cAMP/1%O_2_ induced STC‐1 secretion. A similar interaction between the cAMP/PKA and PI_3_‐Kinase/AKT pathway has been shown in endothelial cells where a cAMP‐mediated inhibition of Rho‐kinase led to an increase in PI_3_‐Kinase activation.^35^, ^36^

Studies in HUVEC and HeLa cells^37^ support the view that both HIF‐1α and HIF‐2α are regulated by mTOR signaling. mTOR is associated with two mTOR complexes, (mTORC) 1 and 2, which act both downstream and upstream of Akt, respectively.^38^ Although HIF‐1α expression seems to be regulated by mTORC‐1 and ‐2, HIF‐2α expression was primarily dependent on mTORC2.^39^, ^40^ To assess the role of mTOR in trophoblast secretion of STC‐1 by 8Br‐cAMP and 1% O_2_, we used rapamycin which targets mTORC1 and KU‐0063794 which inhibits both mTORC‐1 and 2. KU‐0063794, but not rapamycin, significantly inhibited STC‐1 secretion by trophoblasts indicating that mTORC‐2 is primarily involved in the regulation of STC‐1 secretion by BeWo cells.

Activation of mTORC2 in turn activates a number of proteins including Akt, and SGK‐1. In the present study as with Akt, inhibition of SGK‐1 led to a dose‐dependent inhibition of STC‐1 secretion. One of the targets of SGK‐1 is NDRG‐1. NDRG‐1 expression has also been shown to be regulated by hypoxia through the activation of HIF‐1α.^41^ We, therefore, examined the role of NDRG‐1 on the secretion of STC‐1 by BeWo cells. Stimulation of BeWo cells lead to a significant increase in the phosphorylation of NDRG‐1 at both 1% and 21% O_2_ and this was not affected following inhibition of HIF‐2α with PT2385. Direct activation of NDRG‐1 by Dp44tm had no effect on STC‐1 secretion. This suggests that although activated under these conditions, NDRG‐1 played no role in the secretion of STC‐1 by BeWo cells. Both Akt and SGK‐1 are members of the same AGC subfamily of protein kinases and have similar substrate specificities. Both Akt and SGK‐1 phosphorylate and inactivate GSK‐3β. Inhibition of GSK‐3β using kenpaullone resulted in an increase in STC‐1 secretion further supporting a role for GSK‐3β as a negative regulator. When active, GSK‐3β is known to phosphorylate HIF‐1α targeting it for proteasomal degradation.^42^, ^43^, ^44^ Whether GSK‐3β has the same effect on HIF‐2α has yet to be determined.

In conclusion, we have shown that STC‐1 is expressed by syncytial cytotrophoblasts, and is secreted in greater amounts by first trimester chorionic villous tissue under conditions of low oxygen. We have demonstrated that in low oxygen, the secretion of STC‐1 by a human trophoblast cell line is increased by elevated intracellular cAMP. We have further shown that this response is mediated through the activation of the PI_3_‐Kinase/Akt/SGK‐1 pathway primarily through activation of mTORC‐2 and involves the transcription factor HIF‐2α. While some caution should always be taken when extrapolating in vitro results using cell lines to the in vivo situation, we were also able to demonstrate a similar result using chorionic villous explants which more closely model the in vivo situation. This, together with the localization of STC‐1 to the trophoblastic layers of the chorionic villi, points to a potentially important role for STC‐1 in the maintenance of a normal healthy pregnancy.

**Figure 5:**
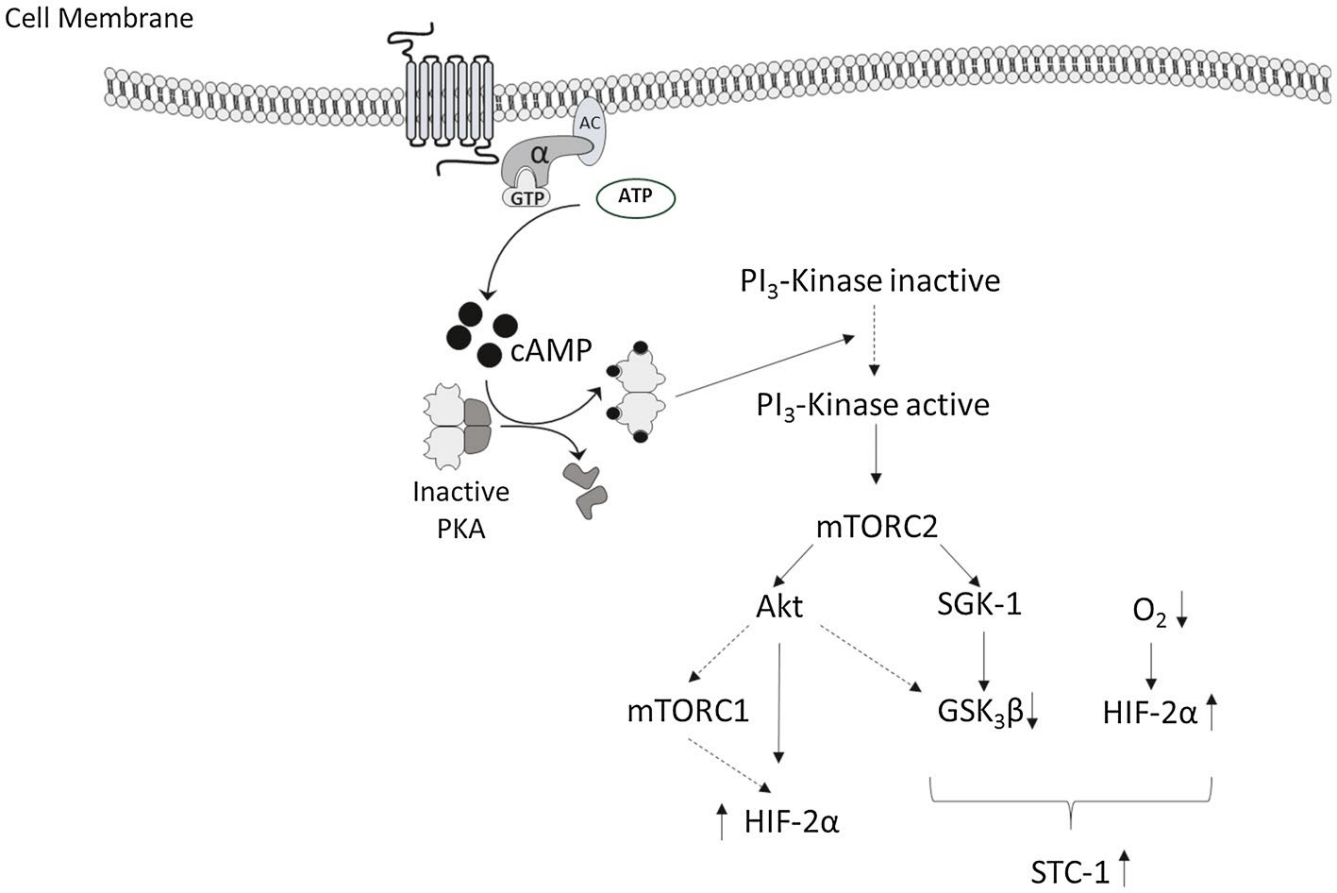
Summary of the pathway intermediates involved in the secretion STC‐1 by BeWo cells. Solid arrows indicate demonstrated connections, dashed arrows are connections based on the literature

Although the role of STC‐1 in pregnancy is unclear it has positive effects on cardiac function including the inhibition of hypoxia‐induced cardiomyocyte apoptosis and attenuation of ischemic cardiac injury. A therapeutic role in heart failure has also been proposed. Given increasing evidence that the maternal cardiovascular system is fundamentally involved in the etiology of preeclampsia,^45^ we postulate that STC‐1 might protect the maternal cardiovascular system from stress in normal pregnancy however its role in preeclampsia/FGR is less clear and warrants further study.

## CONFLICT OF INTEREST

The authors declare no conflicts of interest.

## AUTHOR CONTRIBUTIONS

G.S. Whitley and J.E. Cartwright conceived and planned the study; N. Abid, Z. Tryfonos, J. Bercher, and S.V. Ashton performed the majority of the experiments; B. Thilaganathan and A. Khalil oversaw the recruitment of the patients for the study; G.S. Whitley and J.E. Cartwright wrote the first draft of the paper. All authors contributed to the editing of the manuscript.
